# CCN2/CTGF expression does not correlate with fibrosis in myeloproliferative neoplasms, consistent with noncanonical TGF-β signaling driving myelofibrosis

**DOI:** 10.1007/s00428-024-03799-4

**Published:** 2024-04-11

**Authors:** Roos J. Leguit, Roel Broekhuizen, Moniek de Witte, Reinier A. P. Raymakers, Roel Goldschmeding

**Affiliations:** 1 Dept of Pathology, University Medical Centre Utrecht, H04-3123508 GA, POB 85500, Utrecht, The Netherlands; 2 Dept of Hematology, University Medical Centre Utrecht, Cancer Center, Utrecht, The Netherlands

**Keywords:** Bone marrow, Connective tissue growth factor, CTGF, CCN2, Myeloproliferative neoplasm

## Abstract

The classical *BCR::ABL1*-negative myeloproliferative neoplasms (MPN) form a group of bone marrow (BM) diseases with the potential to progress to acute myeloid leukemia or develop marrow fibrosis and subsequent BM failure. The mechanism by which BM fibrosis develops and the factors that drive stromal activation and fibrosis are not well understood. Cellular Communication Network 2 (CCN2), also known as CTGF (Connective Tissue Growth Factor), is a profibrotic matricellular protein functioning as an important driver and biomarker of fibrosis in a wide range of diseases outside the marrow. CCN2 can promote fibrosis directly or by acting as a factor downstream of TGF-β, the latter already known to contribute to myelofibrosis in MPN.

To study the possible involvement of CCN2 in BM fibrosis in MPN, we assessed CCN2 protein expression by immunohistochemistry in 75 BM biopsies (55 × MPN and 20 × normal controls). We found variable expression of CCN2 in megakaryocytes with significant overexpression in a subgroup of 7 (13%) MPN cases; 4 of them (3 × essential thrombocytemia and 1 × prefibrotic primary myelofibrosis) showed no fibrosis (MF-0), 2 (1 × post-polycythemic myelofibrosis and 1 × primary myelofibrosis) showed moderate fibrosis (MF-2), and 1 (primary myelofibrosis) severe fibrosis (MF-3). Remarkably, CCN2 expression did not correlate with fibrosis or other disease parameters such as platelet count or thrombovascular events, neither in this subgroup nor in the whole study group. This suggests that in BM of MPN patients other, CCN2-independent pathways (such as noncanonical TGF-β signaling) may be more important for the development of fibrosis.

## Introduction

The classical *BCR::ABL1*-negative myeloproliferative neoplasms (MPN) include the subtypes essential thrombocythemia (ET), polycythemia vera (PV), and primary myelofibrosis (PMF). These form an important group of bone marrow (BM) diseases, characterized by the proliferation of cells of one or more of the myeloid lineages and the potential to undergo progression to myelofibrosis or acute myeloid leukemia. BM fibrosis, resulting from the deposition of reticulin fibers and sometimes also collagen fibers, is an important cause of morbidity and mortality in MPN patients as it impairs normal hematopoiesis leading to marrow failure and life-threatening cytopenias.

Previous studies, mainly performed in myelofibrosis models, indicate that the mutant/malignant megakaryocytes contribute to the development of fibrosis by increased expression of fibrotic and pro-inflammatory cytokines and interleukins, growth factors (including transforming growth factor-β (TGF-β)), extracellular matrix components, and other factors [1]. Still, many facets of the development of BM fibrosis and the sequential events that drive stromal activation and fibrosis remain elusive.

**Fig. 1:**
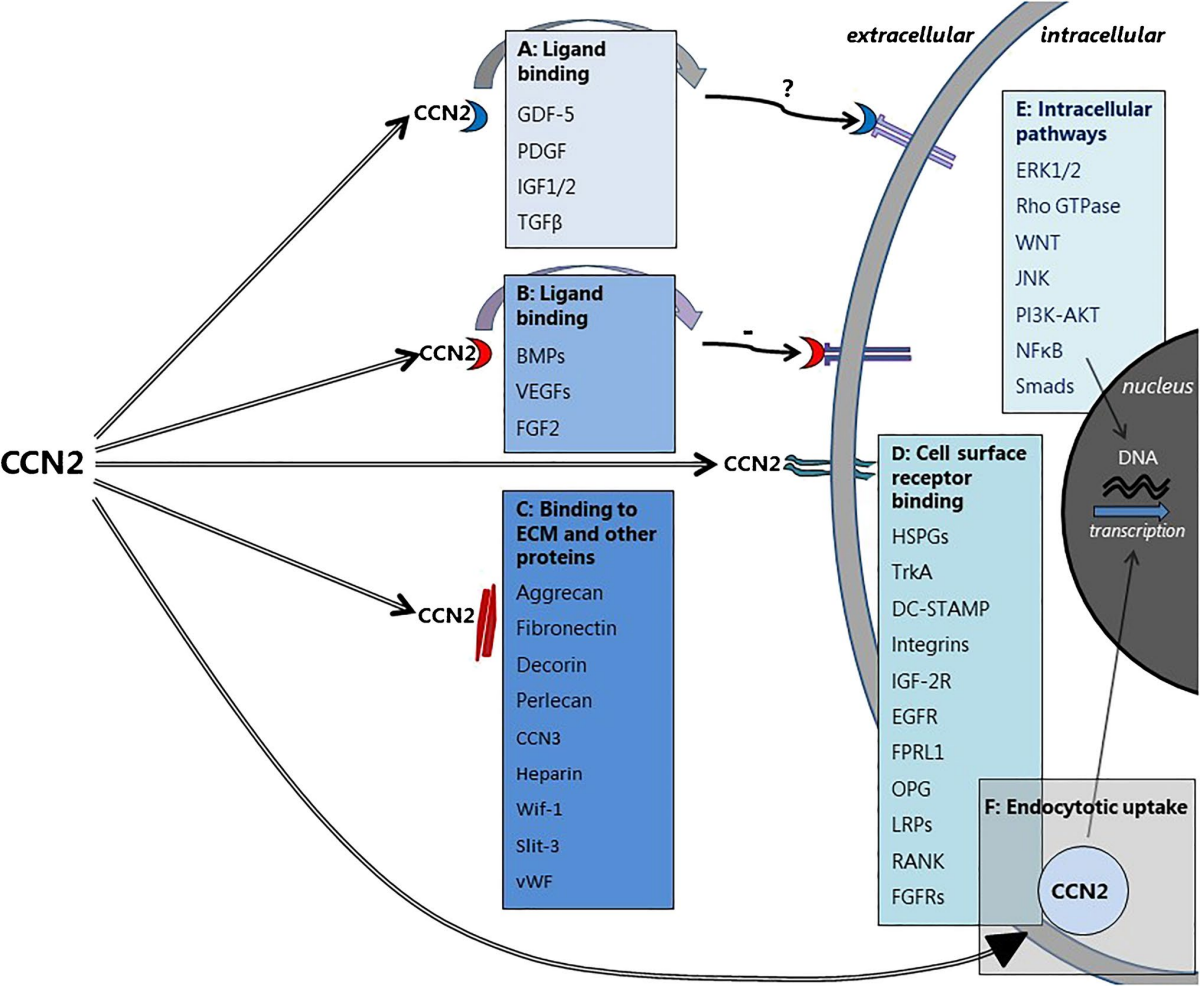
Modes of action of CCN2 (adapted from Leguit et al. J Cell Commun Signal. 2021). Graphic representation of CCN2 interactions and modes of action. For details, please see J Cell Commun Signal. 2021 Mar;15(1):25–56

Due to its critical involvement in many fibrotic processes, a role of Cellular Communication Network 2 (CCN2) in the pathogenesis of BM fibrosis seems likely. CCN2, also known as CTGF (Connective Tissue Growth Factor), is an extracellular matrix cellular protein belonging to the Cellular Communication Network (CCN)-family. It is considered an important driver and biomarker of organ fibrosis in a wide range of diseases [2]. CCN2 is involved in the proliferation, migration, and differentiation of cells and can promote fibrosis directly or by acting as a factor downstream of TGF-β, which is a powerful and well-known inducer of CCN2 transcription [3–8], but many other factors also directly or indirectly induce CCN2 mRNA expression. The mode of action of CCN2 is complex and variable as illustrated in Fig. 1. The way(s) by which CCN2 act(s) on BM target cells is, however, still largely unknown.

In previous studies, CCN2 mRNA expression has been detected in BM mesenchymal stem and stromal cells of normal BM [9, 10]. In addition, altered CCN2 mRNA expression levels have been associated with BM malignancies: in B-acute lymphoblastic leukemia, increased CCN2 mRNA levels are present in B-lymphoblasts, whereas in acute myeloid leukemia, CCN2 mRNA overexpression has been detected in the mesenchymal stem/stromal cells [11, 12]. Furthermore, CCN2 mRNA extracted from BM biopsies of patients with myelofibrosis showed a 27-fold increase when compared to healthy controls, decreasing after allogeneic stem cell transplantation [13]. None of these studies, however, investigated a possible relationship between CCN2 levels and BM fibrosis.

Thus far, CCN2 *protein* expression in the BM has only been investigated in three previous studies [13–15]. Chica et al. found positive staining of several cell populations of normal bone marrow but not in megakaryocytes, except for weak staining near the cell membranes [15], while Åström et al. [14] reported cytoplasmic positivity for CCN2 in a subpopulation (18%) of megakaryocytes in 1 of 5 patients with X-linked thalassemia in almost all (97%) megakaryocytes of all 6 primary myelofibrosis patients, while other hematopoietic cell lineages and the megakaryocytes in normal control BM biopsies were negative [14]. Shergill et al. published an abstract with intriguing observations suggesting a role for CCN2 as a biomarker and a potential target for therapy in MF [13]. However, the abstract (naturally) contained only limited information and statistical detail, and the data presented suggested considerable scatter of data and limited statistical significance. CCN2 staining was found in a higher percentage of megakaryocytes in biopsies of myelofibrosis patients compared to controls (63% vs 40%). This difference did not reach statistical significance (*p* = 0.28), but the mean percentage of CCN2-positive megakaryocytes was significantly higher in myelofibrosis patients at diagnosis (63%) compared to post-transplant biopsies 22% [13]. A correlation between CCN2 expression and fibrosis was, however, not made. Unfortunately, no follow-up publication on this 2015 abstract has appeared to date, and no other studies have since then addressed the role of CCN2 in BM fibrosis. Therefore, we set out to investigate CCN2 protein expression by immunohistochemical staining in a large cohort of 75 BM biopsies (55 MPN patients and 20 normal controls) and correlated the results with the amount of BM fibrosis and other disease parameters.

## Materials and methods

### Patients

To study CCN2 protein expression, we performed immunohistochemistry on in total of 75 BM trephine biopsies, retrieved from the Department of Pathology of the University Medical Center Utrecht, the Netherlands. This cohort encompassed 55 BM biopsies of MPN patients and 20 BM biopsies with normal hematopoiesis. The 55 MPN biopsies consisted of 10 ET cases, 10 post-ET myelofibrosis (post-ET MF) cases, 10 PV cases, 10 post-PV myelofibrosis (post-PV MF) cases, 5 pre-fibrotic PMF (pre-PMF) cases, and 10 cases of PMF presenting in overt fibrotic phase. The 20 normal biopsies had all been obtained as part of the staging procedure for a hematologic neoplasm (mostly diffuse large B-cell lymphomas from immune-privileged sites and 1 case of a MALT lymphoma of the lung), with all patients showing normal blood values and normal hematopoiesis without lymphoma involvement of the BM. The trephine biopsies were formalin-fixed, EDTA-decalcified, and paraffin-embedded. Relevant clinical parameters were extracted from digital patient files.

### Fibrosis

BM reticulin was stained by a silver stain according to Gordon and Sweet, Gomori’s silver impregnation, or by the reticulin stain from DAKO using the Artisan automatic stainer. All reticulin stains were performed on 4 µm thick BM sections. The amount of fibrosis was graded on a scale from 0 to 3, according to the European Consensus of diagnosing bone marrow fibrosis, with 0 being no fibrosis (MF-0), 1 slight fibrosis (MF-1), 2 moderate fibrosis (MF-2), and 3 severe fibrosis (MF-3) [16].

### Immunohistochemistry

Immunohistochemistry for CCN2 was performed on 4 µm BM sections, using primary antibodies from Cell Signaling Technologies (CST) (cat. no 10095S and 86641S), and FG-3114/biotin, provided by FibroGen Inc. All cases were stained with the 10095S antibody. Five 5 MPN cases showing overexpression with the 10095S antibody and 5 normal control cases were additionally stained by the 86641S and FG-3114 antibodies. All slides were deparaffinized and endogenous peroxidase was blocked. For the CST antibodies, antigen retrieval in Tris/EDTA solution at pH 9 was done, followed by primary antibodies (incubation of 86641S for 1 h and 10095S overnight at 4 °C) and detection with BrightVision/HRP anti-rabbit Ig (VWR). As supplied, FG-3114 was conjugated to biotin. Here, endogenous biotin was blocked with a biotin blocking kit (Vector), followed by a primary antibody for 2 h and HRP-labeled streptavidin (Dako). After the HRP labeled reagents, peroxidase was visualized with nova red substrate (Vector labs), followed by a hematoxylin nuclear counterstain.

The intensity of staining was scored semi-quantitatively on a scale from 0 to 3 according to the following staining categories: 0 = no staining, 1 = weak staining, 2 = moderate staining, 3 = strong staining. Contrasting moderate and very strong staining was confined to a subgroup of MPN cases. To highlight this feature, we applied an aggregated weighted score assigning increasing weight to moderate and strong staining patterns. The aggregated, weighted score was calculated as follows: 0 × (score 0) + 1 × (score 1) + 5 × (score 2) + 15 × (score 3).

### Molecular studies

Driver mutations could be retrieved from patient files in 51 of 55 MPN cases. On the remaining 4 MPN cases, NGS was performed using a custom made panel (Ion AmpliSeqTM Haemat Panel) consisting of 43 genes. The presence of non-driver mutations was additionally investigated in the 7 MPN cases with CCN2 overexpression by NGS using the TruSight Oncology 500 (TSO-500) kit, testing 523 genes.

### Statistical analysis

Statistics were done with IBM SPSS 29. Mann–Whitney *U* tests, and linear regression models were used for statistical analysis.

## Results

### Patient characteristics

In total, 75 BM biopsies were stained for CCN2 by the 10095S antibody, of which 55 MPN cases (10 × ET, 10 × post-ET MF, 10 × PV, 10 × p-PV MF, 5 × pre-PMF, 10 × PMF) and 20 control cases with normal marrow. The control group encompassed 15 males and 5 females, with a median age of 63.5 years (range 44–77 years). The MPN group encompassed 32 males and 23 females, with a median age of 60 years (range 16–77 years), being not significantly different from the control group. In the MPN group, 39 cases (71%) contained a *JAK2* V617F mutation, 12 cases (22%) a *CALR* mutation, 2 cases (4%) an *MPL* mutation, 1 case (2%) was triple negative, and in 1 case, the driver mutation could not be determined due to insufficient DNA quality.

### CCN2 expression

**Fig. 2:**
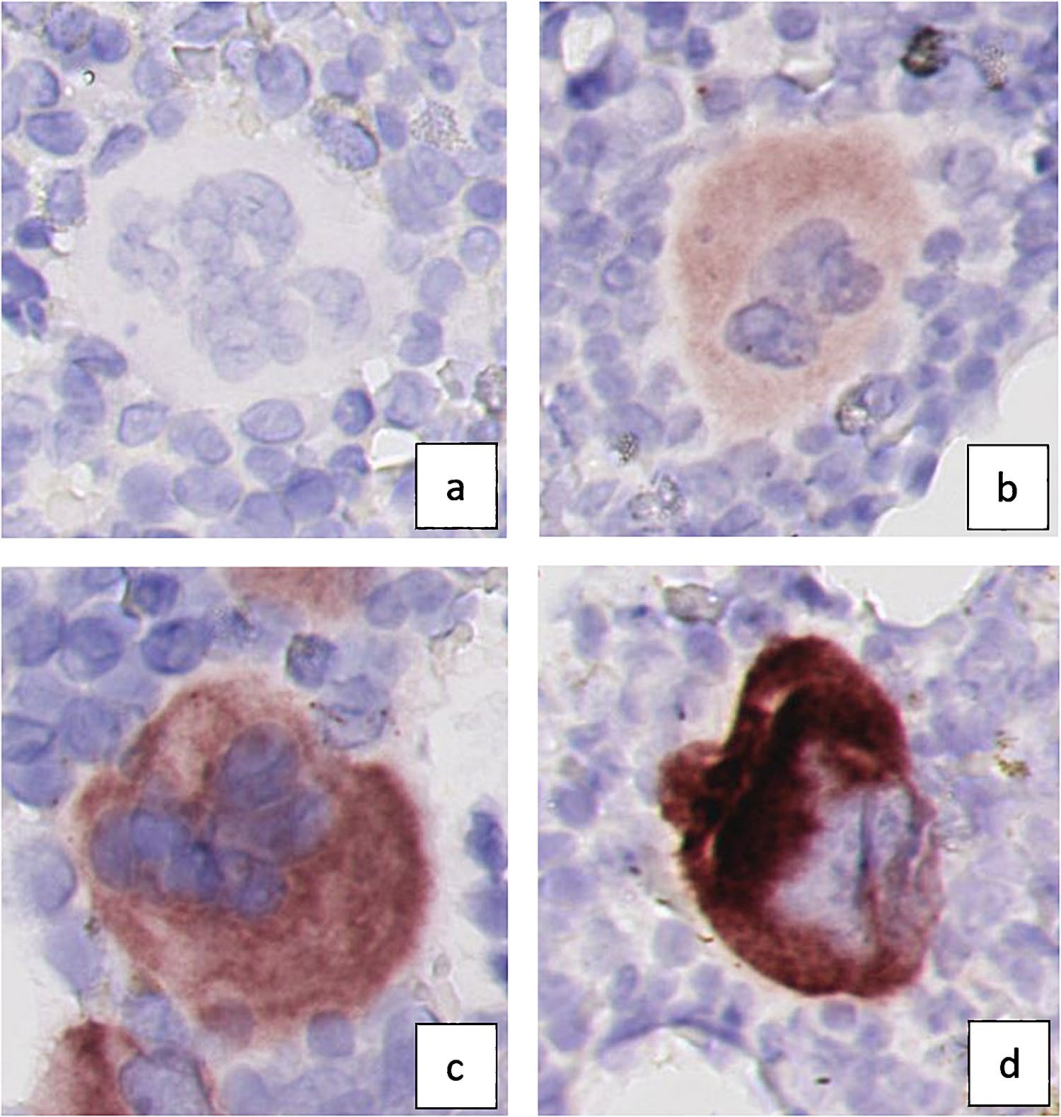
CCN2 scoring of megakaryocytes. Megakaryocytes stained for CCN2 demonstrating **a** no staining (score: 0), **b** weak staining (score: 1 +), **c** moderate staining (score: 2 +), and **d** strong staining (score: 3 +)

When stained for CCN2 by the 10095S antibody, the BM biopsies showed a variable degree of cytoplasmic staining of the megakaryocytes, while the myeloid and erythroid cell lineages were negative. The megakaryocytes showed variable staining, both within and between biopsies. The intensity of staining was scored semi-quantitatively on a scale from 0 to 3 (0 = no staining, 1 = weak staining, 2 = moderate staining, 3 = strong staining) as illustrated by Fig. 2. There was no extracellular staining, which is remarkable as CCN2 is generally considered to be an extracellular protein. Throughout the literature, this appears to be a consistent finding for CCN2 staining, also in other tissues [17, 18], and it was confirmed with 2 additional independent antibodies in the present study. Possible explanations for this finding have thus far largely remained speculative.

**Fig. 3:**
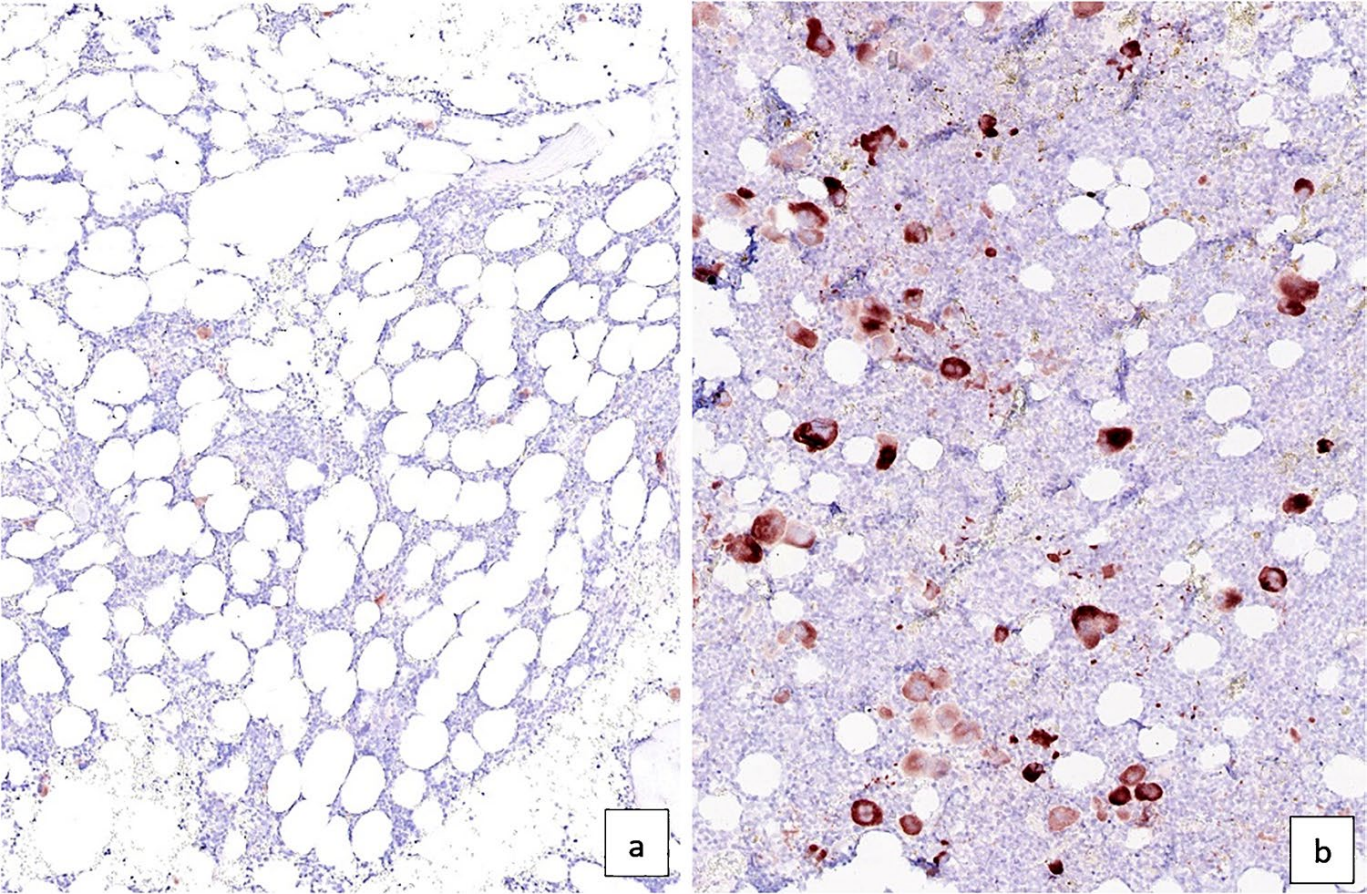
CCN2 immunohistochemical staining of bone marrow biopsies. **a** Normal bone marrow, in which megakaryocytes show no or only weak staining, with only rare megakaryocytes displaying moderate staining. Granulopoiesis and erythropoiesis are negative. **b** An example of a myeloproliferative neoplasm displaying marked overexpression of CCN2 with many megakaryocytes showing moderate to strong cytoplasmic staining

In normal BM, the majority (96–100%) of megakaryocytes in the biopsy showed no or weak staining as illustrated in Fig. 3a. A small number of megakaryocytes (up to 4%) within each biopsy displayed moderate or strong staining, with strongly staining megakaryocytes accounting for at most 2% of the total number of megakaryocytes. The CCN2 expression score ranged from 0.05 to 1.26 with a median score of 0.62.

In MPN, the findings were largely similar, although a few cases (*N* = 7, 13%) clearly stood out by a much stronger staining of the megakaryocytes with a markedly increased number of megakaryocytes displaying moderate or strong cytoplasmic staining, as illustrated in Fig. 3b**.** The moderate/strong staining megakaryocytes could morphologically not be discerned from the negative/weak staining megakaryocytes within the same biopsy. The CCN2 score ranged from 0.00 to 5.90 with a median score of 0.54. The CCN2 score of the MPN group as a whole was not statistically different from the normal control group (*p* = 0.741).

The CCN2 scores of all 75 normal and MPN cases were analyzed by a box-and-whisker plot, which showed 7 outliers (i.e., values greater than 1.5 IQR plus the third quartile), with a significant higher CCN2 expression than the rest of the cases. These cases with a high number of moderate and strong staining megakaryocytes consisted of 3 ET cases (CCN2 scores: 2.82, 3.10, and 3.38), 1 pre-PMF case (CCN2 score: 2.90), 1 post-PV MF case (CCN2 score: 4.65), and 2 PMF cases (CCN2 scores: 2.42 and 5.90).

### Correlation of CCN2 expression with fibrosis

All of the normal BM biopsies and 17 of 75 (23%) MPN cases (10 × ET, 5 × PV, 2 × pre-PMF) showed no fibrosis (MF-0). Mild fibrosis (MF-1) was found in 7 MPN cases (3 × pre-PMF and 4 × PV), moderate fibrosis (MF-2) in 11 MPN cases (7 × post-ET MF, 2 × post-PV MF, 2 × PMF), and severe fibrosis (MF-3) in 18 MPN cases (3 × post-ET MF, 8 × post-PV MF, 7 × PMF). No correlation was found between the CCN2 score and the amount of BM fibrosis (*p* = 0.966).

### Correlation of CCN2 expression with clinical parameters

CCN2 scores did not correlate with age, sex, type of driver mutation, blood values (hemoglobin, leucocytes, platelets, LDH) or the occurrence of thrombovascular events in MPN patients. There was no significant difference in CCN2 score between the different MPN subgroups (*p* = 0.703).

### CCN2 protein overexpression in MPN

In 7 MPN cases (13%), immunohistochemical staining by the 10095S antibody showed significant CCN2 overexpression of megakaryocytes. These were 3 ET cases, 1 post-PV MF, 1 pre-PMF, and 2 PMF cases. Their characteristics are shown in Table 1. Four of them were males and three were females, and the median age was 58 years (range 24–70 years), which was not significantly different (*p* = 0.338) from the 48 MPN cases without CCN2 overexpression (median age 62 years, range 23–77 years). Blood values (hemoglobin, leukocyte counts, platelet counts, LDH) were not significantly different from patients without CCN2 overexpression. Thrombovascular events were as common in MPN patients with CCN2 overexpression (28%) as in those without (27%). Four cases showed no fibrosis (MF-0), 2 showed moderate fibrosis (MF-2), and 1 severe fibrosis (MF-3).

**Table 1:** Myeloproliferative neoplasms with CCN2 overexpression

	Case 1	Case 2	Case 3	Case 4	Case 5	Case 6	Case 7
CCN2 score	2.42	2.82	2.90	3.10	3.38	4.65	5.90
Age	59	45	52	58	24	70	69
Sex	M	F	M	M	F	F	M
Diagnosis	PMF	ET	Pre-PMF	ET	ET	p-PV MF	PMF
Fibrosis	MF-3	MF-0	MF-0	MF-0	MF-0	MF-2	MF-2
Driver mutation (VAF)	*CALR*	*JAK2* (44%)	*JAK2* (28%)	*JAK2* (20%)	*JAK2* (20%)	*JAK2* (79%)	*JAK2* (65%)
Additional pathogenic mutations (VAF)	*DNMT3A* (35%)	*None*	*SF3B1* (32%)	*DNMT3A* (45%)	*TNFAIP3* (54%)	*TP53* (89%)	*CUX1* (6%)
Hemoglobin (mmol/L)	7.1	9.0	9.5	9.3	8.5	4.9	6.3
Leucocytes (× 10^9^/L)	16.8	8.2	10.7	8.4	6.5	30.9	6.3
Platelets (× 10^9^/L)	144	467	757	598	338	72	137
LDH (U/L)	n/a	306	286	316	212	2777	360
Splenomegaly	22 cm	18 cm	n/p	13.4 cm	17 cm	16 cm	22 cm
Signs		Occlusion of retinal artery right eye	Acquired storage pool disease	Erytromelalgia	thrombosis of portal, mesenteric, and splenic vein		
Follow-up	Deceased	Progression to PV	No progression (f-u: 2 yr 10 mo)	No progression (f-u: 1 yr 3 mo)	No progression (f-u: 7 yr 1 mo)	Progression to blast phase (AMKL, p53 mutated) and deceased	No progression (f-u: 1 yr 9 mo)

*ET* essential thrombocytemia, *pre−PMF*prefibrotic primary myelofibrosis, *p−PV MF*post−polycythemic myelofibrosis, *PMF*primary myelofibrosis, *n/p*not palpable, *AMKL*acute megakaryoblastic leukemia, *n/a*not available, *f−u*follow−up

Six of the MPN cases with CCN2 overexpression contained/showed a *JAK2* V617F mutation/clone with a variant allele frequency (VAF) ranging from 5.8 to 79%, not significantly correlating with the CCN2 score (*p* = 0.090). The seventh, one of the PMF cases, contained/showed a *CALR* exon 9 (p.Lys385fs, type II) mutation/clone. The presence of additional pathogenic non-driver mutations was investigated, and an additional mutation was detected in 6 of the 7 cases, being *DNMT3A* (ET and PMF), *NFAIP3* (ET), *TP53* (post-PV MF), *SF3B1* (pre-PMF), and *CUX1* (PMF).

One patient with PMF deceased due to a pneumonia and the patient with post-PV MF deceased after having progressed to a blast phase (in the form of an acute megakaryoblastic leukemia). One patient with ET showed progression to PV. The other patients did not show progression, albeit the follow-up time was limited.

### Staining with additional CCN2 antibodies

To assess whether the CCN2 staining of the megakaryocytes by the 10095S antibody was specific, 5 MPN cases showing overexpression as well as 5 normal BM biopsies were additionally stained by 2 other CCN2 antibodies: FG-3114 and the 86641S antibody. Like the 10095S antibody, these two other CCN2 antibodies also showed cytoplasmic staining of the megakaryocytes. In addition, the aggregated, weighted staining score for the FG-3114 and the 86641S was higher in the 5 selected MPN cases than in the controls, further supporting the notion that the staining with these 3 antibodies indeed reflects CCN2 protein expression.

## Discussion

In this study, we investigated the protein expression of the profibrotic factor CCN2 in BM by immunohistochemistry. We included 20 normal BM biopsies and 55 BM biopsies of MPN patients and correlated the CCN2 staining results with the amount of BM fibrosis and clinical parameters. CCN2 protein expression was detected in a variable degree in the cytoplasm of megakaryocytes, while granulopoiesis and erythropoiesis were negative. High levels of CCN2 expression were seen in 7 (13%) MPN cases, but no correlation was observed between CCN2 expression and fibrosis, neither in the total study group nor in the subgroup with CCN2 overexpression. Thus (megakaryocytic) CCN2 expression appears not to be a key feature in the development of fibrosis in MPN, in contrast to the prominent role that CCN2 plays in the development of fibrosis in many organ diseases outside the marrow [2]. A lack of correlation between CCN2 expression and BM fibrosis is remarkable, especially because the prototypical fibrosis inducer TGF-β, known to also contribute to myelofibrosis in MPN [19], is a potent inducer of CCN2, the two forming a positive feedback loop [4–8].

However, whereas induction of CCN2 gene transcription by TGF-β typically involves the canonical SMAD2/3 pathway [6], a recent study showed that noncanonical c-Jun N-terminal kinase (JNK)-dependent TGF-β signaling in mesenchymal stromal cells is responsible for the development of BM fibrosis in MPN [19]. Our findings may thus be interpreted as further evidence that the non-canonical, rather than the canonical pathway of TGF-β signaling drives BM fibrosis in MPN. This further supports the notion that the exploration of novel modalities for treatment and prevention of BM fibrosis in MPN patients might best focus on the noncanonical pathway of TGF-β signaling.

Interestingly, a subgroup of 7 (13%) MPN cases showed significant CCN2 overexpression. Four of these showed no fibrosis (MF-0), 2 showed moderate fibrosis (MF-2), and 1 severe fibrosis (MF-3), not related to the amount of CCN2 expression. Blood values and clinical signs did not reveal a specific trend. Although CCN2 overexpression was specifically observed in megakaryocytes, no correlation was observed between the level of CCN2 expression and the platelet counts or occurrence of thrombovascular events in this small subgroup, but additional studies on larger cohorts of MPN patients will be needed to better investigate the possible meaning of the megakaryocytic CCN2 overexpression in the development or complications of MPN.

We detected CCN2 protein to be mainly present in megakaryocytes, confirming the findings of previous studies [13, 14]. Chica et al. reported no staining of (normal) megakaryocytes [15], but in the study by Åstrom et al., all their 6 PMF cases showed cytoplasmic CCN2 staining, staining on average 97% of megakaryocytes, while their 6 normal controls were negative [14]. Also, Shergill et al. described variable cytoplasmic staining of megakaryocytes, with a higher percentage of megakaryocytes showing positive CCN2 staining in myelofibrosis patients compared to healthy controls [13]. Discrepancies between the studies might be explained by the use of different types of antibodies. To confirm our results and to rule out non-specific binding of the antibody, we used 2 other CCN2 antibodies binding to different CCN2 epitopes, and the results were in line with our findings with the 10095S antibody.

Shergill et al. found mean CCN2 mRNA levels extracted from BM biopsies in myelofibrosis patients to be 27-fold increase compared to controls, but this difference was not significant. This might suggest that the elevated mean was caused by a small subgroup with very high expression, consistent with our observation of only a subgroup of MPN cases showing marked increased CCN2 protein expression. We have also tried to verify our results at the mRNA level by in situ hybridization on BM sections but did not obtain an interpretable result, probably due to poor mRNA quality after fixation and decalcification. As whole tissue sections were used in the study by Shergill et al., and not isolated megakaryocytes, definite proof that the source of the CCN2 mRNA in BM is the megakaryocytes is still lacking.

Megakaryocytes contain and secrete many factors, by which they contribute to the functioning of the BM microenvironment, including the hematopoietic stem cell niche [1, 20, 21]. They also participate in inflammation and immunity [22]. Also, their role in MPN is well established, in which mutant megakaryocytes play a key role by promoting myeloproliferation and fibrosis [20]. The heterogeneous staining of CCN2 of the megakaryocytes within individual biopsies might reflect different megakaryocyte subtypes, as was previously described [23, 24], or differences between subclones. Morphologically, however, a specific subtype of megakaryocytes showing overexpression could not be detected, and also the degree of CCN2 overexpression did not correlate with the *JAK2* mutant allele frequency. There also was no difference in CCN2 expression between *JAK2* positive and *JAK2* negative MPNs, as opposed to the trend reported in a previous small pilot study [13].

Outside the BM, CCN2 and its fragments have been implicated not only in fibrosis but also in the regulation of cell proliferation, differentiation, adhesion, migration, cell survival, apoptosis, and senescence [25–27]. At least part of CCN2’s biological activity is mediated through interaction with a host of other proteins and receptors, by which it can modify their signaling activity and cross-talk [28–30]. Therefore, it will be challenging to fully explore the potential roles of CCN2 in megakaryocytes of normal BM (although the expression is low), of the subgroup of MPN with overexpression, and beyond.

In summary, in BM biopsies, we observed variable CCN2 expression in megakaryocytes, a cell type increasingly recognized for its importance in the regulation of the BM microenvironment, including the established role of mutant megakaryocytes in MPN promoting myeloproliferation and fibrosis. In MPN, remarkable CCN2 overexpression was detected in a subgroup (13%). CCN2 expression, however, did not correlate with fibrosis or other disease parameters, neither in the whole study group nor in the subgroup with CCN2 overexpression in megakaryocytes.

Our data suggest that CCN2 is not a key driver of myelofibrosis in MPN, and that noncanonical, CCN2 independent, rather than canonical TGF-β signaling might be responsible for the development of fibrosis in MPN.
